# Litigating the Crisis: Towards a Rebalancing of the Rights of Investors Versus Public Interest?

**DOI:** 10.1007/978-3-030-65355-2_12

**Published:** 2021-03-20

**Authors:** Morag Goodwin, Phillip Paiement

**Affiliations:** 1 Tilburg University, Tilburg, The Netherlands; 2 Tilburg University, Tilburg, The Netherlands; 3 Tilburg University, Tilburg, The Netherlands; 4 Tilburg University, Tilburg, The Netherlands; Department of Public Law and Governance, Tilburg Law School, Tilburg, The Netherlands

## Abstract

This chapter reflects on the anticipated rush by private corporations to seek compensation from states for emergency measures taken to address the current health crisis. Where states have, for example, commandeered privately run health facilities, foreign corporations can claim for any negative impact on their current and future profits. This anticipated wave of state-investor litigation draws upon the web of bilateral investment treaties that span the globe and on Investor-State Dispute Settlement (ISDS) mechanisms. In place of a “new normal,” this short paper suggests that these threats represent an intensification of an existing trend, with the main difference being that states of the Global North are increasingly finding themselves disciplined by instruments that they created to protect their own companies abroad. If this happens, as seems likely, this crisis will add to the growing calls to rethink how we regulate the boundary between the public interest and private investors.

This chapter reflects on the anticipated rush by private corporations to seek compensation from states for emergency measures taken to address the current health crisis. Where states have, for example, commandeered privately run health facilities, foreign corporations can claim for any negative impact on their current and future profits. This anticipated wave of state-investor litigation draws upon the web of bilateral investment treaties that span the globe and on Investor-State Dispute Settlement (ISDS) mechanisms. In place of a “new normal,” this short paper suggests that these threats represent an intensification of an existing trend, with the main difference being that states of the Global North are increasingly finding themselves disciplined by instruments that they created to protect their own companies abroad. If this happens, as seems likely, this crisis will add to the growing calls to rethink how we regulate the boundary between the public interest and private investors.

At the heart of this type of transnational litigation is a dispute about the relationship between notions of public and private and the definition of “public interest” that acts as a check on the freedom of action of both states and private actors. The legal basis is the system of Investor-State Dispute Settlement and the complex web of bilateral investment treaties (BITs) and the occasional multilateral trade treaty that constitute the rules of interaction between foreign corporations and investors and the public authorities that host the investment. The system is part of international law and is therefore governed by its general rules. The basic principle of the system is that states protect the interests of companies and investors incorporated in their state when those companies and investors act abroad, by laying down shared—bilateral—rules that prohibit discriminatory action, such as expropriation.

There are many 1000s of BITs currently in operation, most of which were drafted in the 1980s and 1990s. What gives these treaties their bite are the provisions they contain that exclude the domestic courts of the host state from any role in regulating disputes between investors and the state in favor of international arbitration. Yet while BITs are—as the name suggests—a bilateral agreement between two states, it has increasingly been recognized that they are, in practice, unbalanced, and are unfair towards states of the Global South.

In place of a guarantee of mutual interests, BITs have worsened the power imbalance upon which they are based in favor of corporate interests over the public authorities of poorer states (Peronne 2016a, b). The practice of dispute resolution guaranteed by BITs has led to a dramatic expansion of investor rights under the notion of “indirect expropriation,” which covers any measures taken by governments that have a negative impact on investment (UNCTAD 2000). These claims see host governments subjected to claims by foreign firms that frequently result in huge payouts that, in turn, have an equally huge impact on the ability of developing countries to improve public welfare. As such, it has been argued, that the ISDS system promotes the rights of Western-based corporations over those of developing states (Gallacher and Shrestha 2011). This has led to calls for a moratorium on investor-state cases within the ISDS system and a re-writing of the rules of the game (Columbia Center on Sustainable Investment 2020; Kahale III 2018).

## The Current Crisis

The dramatic nature of the policy responses by governments in the face of the COVID-19 pandemic gives rise to a myriad of potential liabilities for states stemming from their obligations towards foreign investors under their international investment agreements. These liabilities concern both public health measures taken to quell the spread and health consequences of the pandemic as well as subsidiary measures taken in response to the pandemic’s social and economic consequences, including those flowing from corresponding public health restrictions. Examples of direct public health measures that may pose harmful consequences to the undertakings of foreign investors include: the nationalization of private medical care facilities; compulsory licensing for patented drugs and medical devices; the reduction or prohibition of the exportation of medical materials; requisitioning hotels to serve as health clinics or quarantine sites; and compelling manufacturers to produce medical supplies.

In addition, the following social and economic policies intended to limit the consequences of the pandemic could also impact foreign investors in a manner that would give rise to ISDS arbitration: moratoriums on toll-roads, rent payments, and utilities payments; the introduction of limitations on contractual liabilities and other creditor protections; limitations of foreign investments to protect against buyouts of distressed assets; moratoriums on bankruptcy proceedings; suspension of mortgage payments; moratoriums on utilities connection cancelations; tax relief measures to support businesses particularly affected by the pandemic; and the prohibition of companies domiciled in tax havens from receiving financial aid. In a “damned if you do, damned if you don’t” manner, states might even be exposed to liability if they fail to take sufficient measures to ensure public order. Based on the concept of indirect expropriation, such governments could face claims if looting or other social unrest affects foreign investors, as was the case in Egypt during the Arab Spring when disruptions and theft from gas pipelines led Ampal-American Israel Corporation to initiate ISDS proceedings against Egypt for $535 million, a procedure which is still pending. It is quite possible to imagine ISDS claims arising both for a failure by governments to prevent a subsequent wave of infections due to a lifting of restrictions too quickly or because they failed to enforce restrictions sufficiently well; and because they implemented lockdown rules in the first place. Simply put, the complexity of policy responses to the pandemic presents countless opportunities for ISDS to arise.

Already in late March, while the pandemic was in its worst stages in Europe with thousands of deaths each day, attorneys from ArbLit in Milan were publicly contemplating the potential investment claims that may come out of the pandemic (Benedetteli et al. 2020). Since then, numerous law firms specialized in investment arbitration have been contacting potential clients in order to raise awareness about the types of claims that they may be able to pursue against states whose measures have impacted their businesses.

## Fighting Back

Despite it long being accepted by many scholars, countries, and international institutions that the ISDS system is detrimental to the interests of the Global South, the system perseveres. Previous government crises, such as Argentina’s 15 year-long financial crisis of 1998–2002, or the Arab Spring—while they led to calls for reform—did not lead to change. Nor has the permanent governance crisis of many Global South states created sufficient momentum for a rebalancing of investors’ rights in favor of the public interest. The system is not unchallenged, however. Bolivia, Ecuador, and Venezuela have all withdrawn from the International Centre for Settlement of Investment Disputes (ICSID) Convention this century (in 2007, 2009 and 2012, respectively) (Voon and Mitchell 2016). Other efforts to fight back include the termination of older generation BIT agreements: for example, Venezuela terminated its BIT with The Netherlands in 2008 after it was used as the basis for ICSID jurisdiction in 10 cases (Fach Gómez 2010), Bolivia has terminated 10 of its 23 BITs, and Indonesia has terminated 26 of its 71 BITs (Ranjan 2019).

However, the fight back is not limited to states of the Global South. In recent years, a number of wealthy countries have recognized that the system does not serve their interests either. In 2009, the Australian government requested the Productivity Commission—a governmental advisory body—to investigate the impact of Australia’s bilateral and trade agreements on Australia’s economic performance. Its 2010 report found no evidence that ISDS provisions increase foreign investor inflow and held that, in contrast, ISDS provisions represented “considerable policy and financial risks” to the Australian state. Its conclusion, that “the Australian government should seek to avoid the inclusion of investor state dispute settlement provisions in bilateral and regional trade agreements,” led to a rethink of Australian trade policy (Productivity Commission 2010; Ranald 2011). Notably, the report immediately preceded a lengthy and costly ISDS claim filed by tobacco conglomerate Philip Morris’s Hong Kong subsidiary against Australia for its 2011 tobacco plain packaging legislation, a dispute in which Australia ultimately prevailed, paving the way for similar legislation in developing countries.

Similarly, the Dutch government decided in 2018 to create a new BIT model treaty to serve as the basis for the renegotiation of all 79 BITs between the Dutch state and non-EU countries. The new model is designed to create a fairer balance by introducing stricter eligibility criteria for investors to be able to make claims against states. One significant change is that claims of indirect expropriation will now require “fundamental attributes of property” to be taken, which means, for example, that fluctuations in prices as a result of government measures will no longer be claimable (Marsman 2018). These changes by the Dutch government are important, as Dutch BITs are the second most invoked by investors globally, and the old model is widely viewed as offering “the gold standard” of investor protection. This change of heart by the Dutch government is prompted by recognition of the need to rebalance the rights of investors against state interests. More importantly, it follows the realization that power shifts in the global economy entail that the Netherlands—traditionally an investment-exporting country—is now primarily a capital- importing state. For example, 36% of residential rental properties sold in the Netherlands in 2019 were purchased by foreign investors (Rachid 2019). Strict investor protection is increasingly viewed by the Dutch state as detrimental to its interests in place of protecting them (Duggal and van de Ven 2019). It is not alone amongst Northern states.

## Litigating This Crisis

Should states be faced with investment arbitration in response to their COVID-19 policy measures, they have two general pathways to defend their actions (Martinez 2010). First, they can rely on the defense of necessity as developed in customary international law. However, exceptions under necessity or emergency scenarios are not usually recognized in international investment agreements, which raises the risk that an arbitration panel will not accept such a defense. Second, they may rely on regulation in the public interest exception clauses, which are increasingly common in international investment agreements, yet fairly uncommon in older agreements. This justification, if available, would be subject to proportionality testing by an arbitration panel to determine whether measures less harmful for foreign investors would have been possible. There is a fair chance that many states could successfully defend their actions in this manner. Yet the prospect of proportionality evaluations and potential massive payouts to foreign investors for government actions in a pandemic that has taken the lives of hundreds of thousands of Europeans and North Americans is likely to be deeply unpalatable to these governments and their voters. We should not anticipate that actions by corporations in response to the pandemic could lead to the development of a “new common” amongst states—competition is, after all, the beating heart of capitalism and hence of the global economy. The most that can be hoped is that the pandemic gives powerful states the incentive to initiate reforms to the international investment treaty system and hence continue the trend towards a rebalancing of public interest and investors’ rights.
